# Granulocyte colony-stimulating factor for recurrent implantation failure: the results of a 5-years cohort study

**DOI:** 10.1038/s41598-025-24587-3

**Published:** 2025-11-18

**Authors:** Islam Elkhateb, Radwa Fahmy, Wafaa Ramadan, Ahmed Mourad, Nihal M. El-Demiry, Amal Shohayeb

**Affiliations:** 1 Department of Obstetrics and Gynaecology, Students’ Hospital, Cairo University, Cairo, Egypt; 2 Department of Obstetrics and Gynaecology, Kasr Al-Ainy Hospital, Cairo University, Cairo, Egypt; 3 Department of Dermatology, Kasr Al-Ainy Hospital, Cairo University, Cairo, Egypt

**Keywords:** Granulocyte colony-stimulating factor, Filgrastim, Assisted reproductive techniques, Embryo implantation, Pregnancy rates, Diseases, Health care, Medical research

## Abstract

Despite the significant progress in the management of infertility, recurrent implantation failure (RIF) is yet a challenge for infertility physicians. It has a deep financial and psychological impact on RIF patients. Granulocyte-colony stimulating factor (G-CSF) has emerged as a chemical that can increase embryo implantation and clinical pregnancy rates in patients with RIF. This ambispective observational cohort study was carried out at the IVF unit of Cairo University Kasr Alainy Obstetrics and Gynaecology Hospital between October 2019 and September 2024. All RIF patients who underwent fresh embryo transfer (ET) from 10/2019 to 9/2023 were treated with intrauterine (IU) infusion of G-CSF after oocyte pick-up and during the mock ET test, three days before fresh ET. The outcomes of RIF patients who had fresh ET before and after these dates were recorded and used as the control group. We excluded patients who were very old in age (> 42 years old) as well as those who had significant uterine, immunological or genetic factors that might affect implantation. We analyzed 40 patients in the study group and 39 in the control group. The study group had higher IU clinical pregnancy (27.5% vs. 17.9%) and chemical pregnancy rates (35% vs. 23%), but neither was statistically significant (*P* = 0.28 and *P* = 0.243, respectively). The implantation rate was significantly higher in the study group (0.33 ± 0.25 vs. 0.33 ± 0.08, *P* = 0.04). First-trimester abortion was lower in the study group (18.2% vs. 42.9%), but not statistically significant (*P* = 0.25). Endometrial thickness increased by 1.15 ± 1.5 cm in the study group; data for this variable in the control group were poorly documented. IU G-CSF administration in idiopathic RIF patients before undergoing fresh ET improved implantation rates, while it did not alter clinical or chemical pregnancy or first-trimester abortion rates.

**Trial registration**: This study was registered with the identifier NCT04998279 on 26/7/2021 in the National Library of Medicine registry (clinicaltrials.gov).

## Introduction

Only 20–25% of fertile couples achieve pregnancy in one menstrual cycle^1^. In an in vitro fertilization (IVF) cycle, the chance of pregnancy does not exceed 40%. It is assumed that implantation failure occurs in some of the cycles that do not result in pregnancy^2^. 25% of pregnancies fail even before a woman misses her period^3^. Implantation failure has been defined as the failure of the embryo to produce an ultrasound-visible intrauterine (IU) gestational sac, whether it was associated with a positive pregnancy test (chemical pregnancy) or not^2^. Implantation failure is reported to represent 75% of pregnancy losses^4,5^. Since a single implantation failure often goes unnoticed or can have multiple causes in couples seeking conception, recurrent implantation failure (RIF) was more meaningful to investigate and manage.

RIF had been defined as the absence of implantation after two consecutive IVF or frozen-thawed transfer cycles where at least four cleavage-stage or two blastocyst-stage good-quality embryos were transferred in total^6^. A more recent description was given by the European Society of Human Reproduction and Embryology (ESHRE) as “the scenario in which the transfer of embryos considered to be viable has failed to result in a positive pregnancy test sufficiently often in a specific patient to warrant consideration of further investigations and/or interventions”^7^. Embryonic, immunological, thrombophilic, lifestyle, and uterine and tubal anatomic factors can cause RIF^2^. With advanced technology and management of these factors, reproductive endocrinologists grasped the two key elements in RIF: the embryo and the endometrium^8,9^. An accommodating and healthy endometrium allows endometrial cells to convert into decidual cells, promotes blastocyst implantation, and aids in the swift progression of placental development^10^. Research trials have shown that immune cells, growth factors, cytokines, and hormonal changes can improve implantation rates^2,7,11–13^.

Granulocyte-colony stimulating factor (G-CSF) is a glycoprotein cytokine that is produced by bone marrow cells, stromal cells, fibroblasts, and macrophages^14^. G-CSF is also produced in the maternofetal interaction during embryo implantation in early pregnancy, suggesting that it may be involved in decidua and placental functionality^15^. G-CSF receptor expression increases throughout pre-ovulatory follicle maturation, in human endometrium, and in luteinized granulosa cells^16^. G-CSF facilitates endometrial regeneration by promoting angiogenesis and decreasing cell death by reducing apoptotic activity^17^. G-CSF plays a role in embryo implantation and the continuation of pregnancy by temporarily suppressing immune response through its effects on lymphocytes, macrophages and T helper-2 cells^18^. Thus, G-CSF supplementation has been considered as a promising innovative therapy in reproductive medicine.

Previous studies have investigated the use of G-CSF in patients with recurrent miscarriage^19^, resistant thin endometrium^12,15,20–24^, normal IVF patients^16,25^, and RIF patients^8,9,26–32^. Based on low-quality and conflicting evidence, meta-analyses of these studies reported a positive effect on reproductive outcomes in recurrent miscarriage, thin endometrium, and RIF patients but not on normal IVF patients^17,33–42^. In our study, we aim to contribute to providing high-quality evidence of G-CSF use via IU infusion to improve reproductive outcomes in idiopathic RIF patients undergoing fresh IVF cycles by reporting the results of a well-designed cohort study conducted at a well-known tertiary care fertility center in a low and middle-income country [LMIC].

## Methodology

This ambispective (prospective and retrospective) cohort study was conducted between October 2019 and September 2024 at the IVF Unit of the Obstetrics and Gynaecology (OBGYN) Department at Cairo University Kasr Alainy Teaching Hospital in Egypt. The Cairo University Faculty of Medicine Research Ethics Committee approved it with the code MD-179-2019. The OBGYN Department Research Ethics Committee approved the ambispective design, which involves both retrospective and prospective data collection. Our research was performed in accordance with the relevant guidelines and regulations. It was registered with the identifier NCT04998279 on 26/7/2021 in the National Library of Medicine registry (clinicaltrials.gov). All patients had full counselling and signed a written informed consent before participating in the study.

### Patient selection

Eligibility criteria included any infertile patient in reproductive age with two or more consecutive failed IVF trials with the transfer of at least two grade A cleavage stage embryos or one blastocyst stage embryo in each cycle, and the availability of more than 3 M2 oocytes after induction of ovulation (IOO) and ovum pick-up (OPU) in the investigated cycle. Patients with uncontrolled medical, autoimmune, or thrombophilia disorders; those with contraindications to G-CSF (such as a history of malignancy, sickle cell disease, chronic neutropenia, congenital fructose intolerance, chest infection, or renal failure); and patients with genetic disorders were excluded from our study. The final analysis did not include patients with any factor that could affect the endometrial implantation. This includes patients > 42 years old and patients with endometriomas > 4 cm, hydrosalpinx, uterine anomalies, or persistent endometrial pathology such as submucous myomas, endometrial polyps, synechiae, persistent thin endometrium (less than seven millimetres), and endometritis. The final analysis did not include patients in which fresh embryo transfer (ET) was cancelled due to non-viability of embryos after OPU or was done with embryos other than at least one grade A embryo, patients who received G-CSF before frozen-thawed ET cycles as freeze all embryos decision was taken after OPU due to early signs of ovarian hyperstimulation or an endometrial pathology, and patients whose files showed poor documentation that the eligibility criteria could not be ensured.

### Intervention protocol

All eligible RIF patients treated in the unit between 10/2019 and 9/2023 were included in the study group. At our unit, every patient is assessed by taking a complete record of her obstetric, menstrual, medical, surgical, infertility, and previous trials history (if any). A pelvic examination then follows, with taking her vitals and weight. A transvaginal sonography and complete hormonal profile are carried out on day 2 of menses, along with the routine haematological, biochemical, coagulation, and other labs if indicated. The husbands undergo semen analysis at our unit. All findings are then presented at the weekly staff meeting, and a decision is made regarding the IOO protocol and/ or any other pre-IOO corrective measures. According to the patient assessment, IOO follows either the fixed antagonist, long agonist, or short agonist protocols, and ovarian stimulation is performed with daily intramuscular injections of 75–450 IU of Human Menopausal Gonadotropins (Merional, IBSA, Switzerland; Fostimon, IBSA, Switzerland). Ovarian response is monitored using transvaginal sonography until at least three follicles reach 18–20 mm. Then, ovulation is triggered by Human chorionic gonadotropin (HCG) (Choriomon, IBSA Pharmaceutical, Switzerland) or a gonadotropin-releasing hormone agonist (Decapeptyl, Ferring Pharmaceuticals, Germany) in some cases under the antagonist protocol. Transvaginal sonography-guided OPU is performed under general anaesthesia 34–36 h later. After OPU, if more than 3 M2 oocytes are retrieved, then an IU ultrasound-guided infusion of 300 µg (1 mL) of recombinant human G-CSF (Filgrastim, Sedico, Egypt) is carried out through an IU insemination [IUI] catheter during the routine mock ET test in the study group, while it is carried out without any injections for patients in the control group. Three days later, ultrasound-guided fresh ET of only good-quality embryos (grade A embryos) is performed using a flexible ET catheter (Cook Medical LLC, USA). Luteal support is provided with progesterone 400 mg/twice per day (Prontogest, Marcyrl, Egypt) from oocyte retrieval until the pregnancy test. For the control group, all eligible RIF patients who underwent new IVF trials at our unit from January 2016 to October 2019 and from September 2023 to September 2024 were investigated and included.

### Assessment of outcomes

The endometrial thickness is measured on the day of G-CSF injection and the day of ET to assess the (difference in endometrial thickness). The serum HCG test to detect pregnancy is performed on day 14 after ET. If positive (chemical pregnancy), then the woman undergoes TV-US after 2 weeks to visualize the IU gestational sac and viability of the embryo (clinical pregnancy rate (CPR); the primary outcome). If positive, then the implantation rate (IR) (number of gestational sacs/ number of embryos transferred) is calculated, and patients are followed to the end of the first trimester to detect the (first-trimester abortion rate).

All variables, such as patient demographics and baseline clinical criteria, male factor, IOO regimens, M2 oocytes retrieved, endometrial thickness difference, grade A embryos transferred, chemical pregnancy, CPR, IR, abortion rates, and any observed G-CSF side effects were recorded and compared between the two groups.

### Sample size calculation

The sample size calculation was based on our primary outcome, the CPR. The calculation was done by comparing two independent proportions from independent samples in a prospective study using the Chi-square test. The α-error level was fixed at 0.05, the power was set at 80%, and the ratio of unexposed to exposed in the sample was set at 1. As previously published^43^, the CPR in the G-CSF-treated group was 50.7%, and 19.8% in the control group. Accordingly, the minimum optimum sample size should be 36 patients in each group. 10% (4 patients) were added to compensate for any potential drop-out or exclusion. Sample size calculation was done using the Clincalc online statistical calculator^44^.

### Data analysis

Data of categorical variables were summarized as frequencies and percentages and compared using the chi-square test. We used the Shapiro-Wilk P test to test the normality of quantitative data distribution. As most of the data were not normally distributed (Shapiro-Wilk *P* < 0.05), data were summarized as median and interquartile ranges [IQR] and compared using the Mann-Whitney U test. The statistical significance was considered as *P* < 0.05. Statistical analysis was performed using Jamovi software, version 2.3.28 for iOS. The study was reported using the STROBE standard checklist for reporting cohort studies.

## Results

**Fig. 1:**
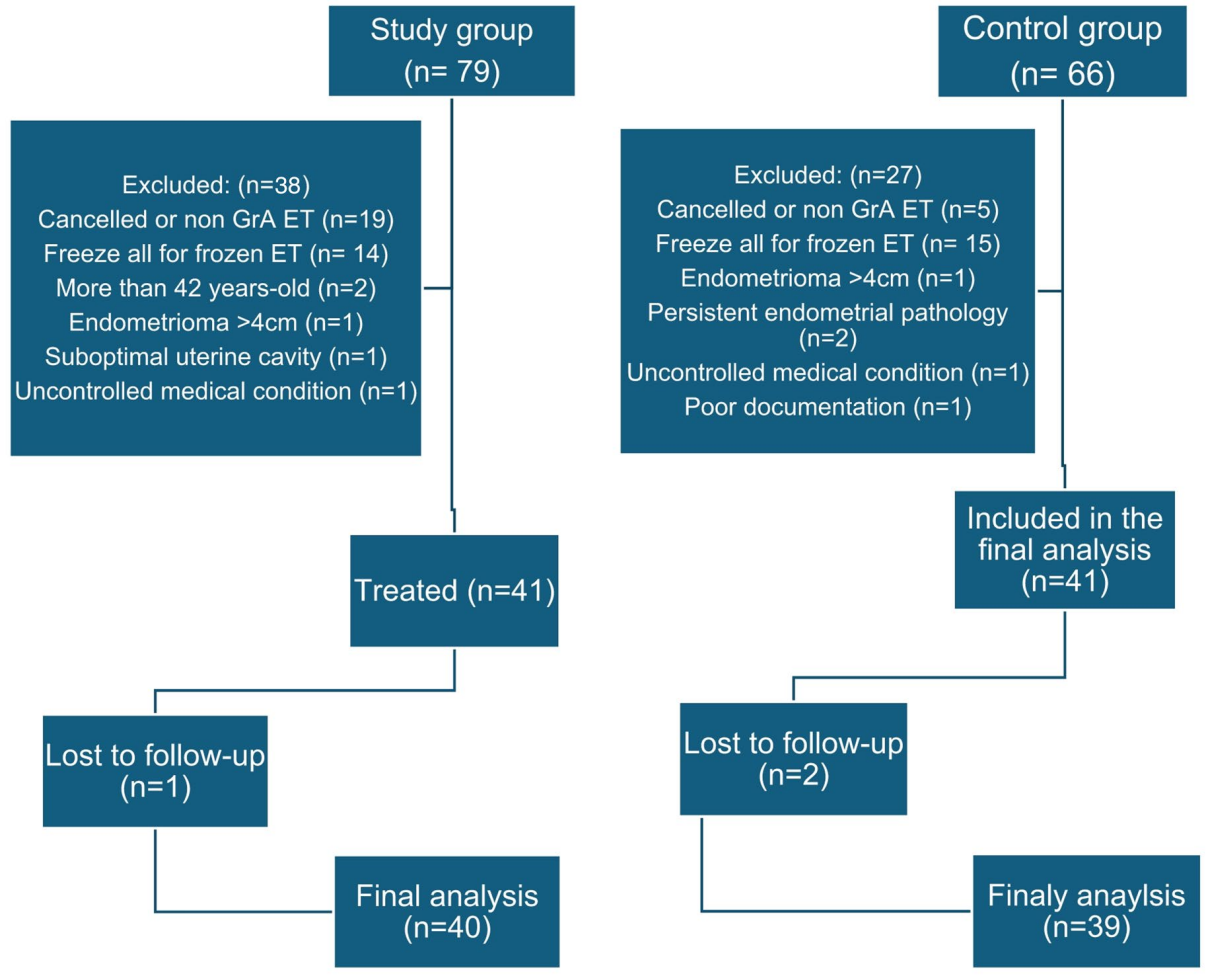
STROBE flowchart of the study.

Between October 2019 and September 2023, 79 patients were treated with G-CSF after OPU. In 19 patients, ET was cancelled due to the non-viability of embryos or was done using grade B or C embryo/s. In 14 patients, a freeze-all and frozen-thawed ET decision was taken due to signs of ovarian hyperstimulation or persistent endometrial pathology visualized after OPU, such as a polyp. Two Patients were 43 and 45 years old. One patient had an endometrioma > 4 cm. One patient had high-risk thrombophilia and a suboptimal cavity due to previous uterine surgeries. One patient had uncontrolled diabetes and systemic lupus erythematosus with kidney disease. One patient lost to follow-up after G-SCF treatment and ET. For the control group, we reviewed the files of 66 eligible patients. In 15 patients, a freeze-all and frozen-thawed ET decision was made. In five patients, ET was cancelled due to the non-availability of embryos or was done using grade B or C embryo/s. Four patients had confounding endometrial, adnexal, or medical factors, one had very poor documentation, and two had lost to follow-up. (Fig. 1) Finally, we analyzed the results of 40 patients in the study group and 39 in the control group. There was no significant difference between the study and control groups regarding the demographic variables and induction of ovulation parameters. (Table 1)

**Table 1:** Demographic and induction of ovulation variables of the study patients.

Criteria | Group		Study group (*n* = 40) Median (IQR)	Control group (*n* = 39) Median (IQR)	*P*- value
Age (years)		32.5 (8.25)	34 (5)	0.417
BMI		29 (6.25)	Valid: 38 30 (8.13)	0.255
Type of infertility	Valid	40	37	
	Primary	21	14	03197
	Secondary	19	23	0.197
Duration of infertility (years)		6 (6)	Valid: 37 7 (6)	0.387
Previous ICSI trials		2 (1)	2 (1.5)	0.707
Severe male factor		7/ 40 (17.5%)	4/39 (10.2%)	0.352
IOO protocol	Antagonist	9	13	0.283
	Long agonist	24	22	0.947
	Short agonist	7	4	0.352
Cycle duration (days)		13 (3)	14 (2)	0.058
Gonadotrophin dose/ day		300 (75)	300 (113)	0.519
M2 oocytes retrieved		5 (4)	5 (3)	0.266
Grade A embryos transferred		3 (1)	3 (2)	0.603
Endometrial thickness on OPU (mm)		Valid: 36 10 (1.35)	Valid: 29 10 (3.35)	0.582
Endometrial thickness on ET (mm)		Valid: 36 11 (2)	Valid: 6 9.5 (4.1)	0.328
Observed side effects		0	NA	NA

BMI: Body mass index. ET: Embryo transfer. ICSI: Intracytoplasmic sperm injection. IOO: Induction of ovulation. OPU: Ovum pick-up.

The clinical outcomes are summarized in Table 2. Even though the IU CPR, chemical pregnancy and IR were higher in the study group than in the control group, and the first trimetric abortion rate was lower, but it was only statistically significant for the IR. (P-value: 0.04). While the study group had a 1.15 cm+/- 1.5 cm increase in endometrial thickness from the day of oocyte pick-up to the day of ET, poor documentation of this outcome in the control group made it impossible for a comparison. (Table 2). There was one ectopic pregnancy case in the study group that was not considered a clinical pregnancy or a first-trimester abortion, as the pregnancy was terminated. No side effect was observed for the study group.

**Table 2:** Results of the study (primary and secondary outcomes).

Criteria | Group	Study group (*n* = 40)	Control group (*n* = 39)	*P*- value
Intrauterine clinical pregnancy	11/40 (27.5%)	7/39 (17.9%)	0.28
Chemical pregnancy	14/ 40 (35%)	9/39 (23%)	0.243
Implantation rate	0.33 +/- 0.25	0.33 +/- 0.08	0.04
First- trimester abortion	2/11 (18.2%)	3/7 (42.9%)	0.25
Endometrial thickness difference	Valid: 36 + 1.15 +/- 1.5 cm	–	–

## Discussion

### Introduction | gaps in previous research

In the scientific literature, RIF has received less attention than recurrent miscarriage or poor ovarian reserve [POR], a remarkable cause for recurrent IVF failure. RIF has multiple definitions that are enlisted by Polanski et al. in 2014^6^. These definitions vary according to the number and treatment modality used in previous failed cycles, the number and quality of embryos transferred, with some definitions incorporating additional criteria such as maternal age, embryo stage, and the absence of other female factors. In 2023, the ESHRE described RIF as “the scenario in which the transfer of embryos considered to be viable has failed to result in a positive pregnancy test sufficiently often in a specific patient to warrant consideration of further investigations and/or interventions”^7^. While this description is more adaptive and case-senstive with no cut-offs for the number of previous trials or embryos transferred, our study started four-years prior to this ESHRE good practice recommendations on RIF. In our study, we used the definition concluded by Polanski et al. in 2014 after analyzing RIF definitions from 44 studies, which is “ absence of implantation after two consecutive cycles of IVF, ICSI or frozen-thawed embryo replacement cycles where the cumulative number of transferred embryos was no less than four for cleavage-stage embryos and no less than two for blastocysts, with all embryos being of good quality and of appropriate developmental stage”^6^.

Although meta-analyses of previous studies done on G-CSF use in patients with RIF in fresh ET cycles via the IU^8,9,26,27^, and subcutaneous (SC) routes^28–31^, and in frozen-thawed ET cycles^31,32^, reported a beneficial effect^17,33–35,37–40^, clinical guidelines do not yet recommend its use for RIF due to the limited and low-quality evidence from these studies^7,11^. In our study, we aim to contribute towards high-quality evidence of G-CSF use in RIF patients in the specific setting of IU administration before fresh ET. This is by reporting the results of a 5-year cohort study on carefully selected idiopathic RIF patients. The control group received a mock ET test after OPU to have the same endometrial injury effect as the study group. Both groups were treated at the same unit using the same lab equipment and by the same physicians. This consistent setting helped minimize confounding factors and ensured that any observed differences in outcome could be confidently attributed to the G-CSF treatment. Even though it is a single-center study with a relatively small sample size, it can be combined and analyzed with similar studies in meta-analyses to generate stronger evidence.

### Summarizing key findings

In our study, G-CSF was associated with improved reproductive outcomes in patients with idiopathic RIF and normal endometrium using the IU infusion route in fresh ET cycles. This was statistically significant for IR and maybe clinically significant for IU CPR, chemical pregnancy, and first-trimester abortion rates. No side effects have occurred to any patient in the study group.

### Interpreting results| compare with other studies

Previous studies on G-CSF use versus placebo via IU infusion in RIF patients undergoing fresh ET showed some heterogeneity^8,26,27^. Kalem et al. and Davari-Tanha et al. defined RIF patients by the failure of three or more IVF cycles where at least four good-quality embryos were transferred in total^8,27^. Eftekhar et al. definition was closer to the one we used and they defined it by the failure of two implantations^26^. Similar to Eftekhar’s study^26^, we transferred embryos at day three (cleavage stage), while embryos were transferred at day three or five in Kalem and Davari-Tanha’s study^8,27^. In Kalem’s study, G-CSF was infused on the day of HCG trigger^8^, while in Davari-Tanha, Eftekhar, and our study, it was given on the same day of OPU^26,27^. Only in Eftekhar’s study was 0.5mL (300 mcg) of G-CSF infused IU^26^, whereas in Davari-Tanha, Kalem, and our study, 1mL was administered^8,27^. Regarding the results, while Eftekhar’s study reported statistically significant improvements in IR and CPR^26^, Davari-Tanha and our study found only a statistically significant improvement in IR. It is worth noting that Davari-Tanha’s study included 15% frozen-thawed ET cycles in their final analysis^27^. Only in Kalem’s study, the authors did not find any statistically significant results^8^. In our opinion, infusing the G-CSF very early during the HCG trigger day in Kalem’s study might have affected their findings.

SC G-CSF use in RIF patients during fresh ET cycles has been studied by Aleyasin, Arefi and Scarpellini, and they all reported beneficial effects, though the significance of this effect varied^28–30^. In meta-analyses, the SC route was found to be more effective than the IU one^33,39^, with one analysis reporting that SC is the only effective route^35^. In our study, we were keen on minimizing the systemic side effects of G-CSF, which maybe more common with SC route^7,11,42^, and we basically started our study before these analyses findings were available.

For G-CSF use before frozen-thawed ET, IU G-CSF use has been reported to statistically reduce the miscarriage rates and clinically improve the CPR and live birth rates in one study^32^. In another study that compared G-CSF use via SC or IU routes versus placebo before frozen-thawed ET, the SC route provided better results than the IU infusion, and both were better than placebo^31^. Meta-analyses have reported that G-CSF improves reproductive outcomes in fresh and frozen-thawed ET via both routes^33,37,39^. At our unit, freeze all with later frozen-thawed ET decision is taken only for patients with signs of ovarian hyperstimulation or endometrial pathology diagnosed at/ after OPU. We are currently running another study on IU G-CSF use before frozen-thawed ET for these patients, and we are witnessing significant results in favour of G-CSF.

Finally, previous studies have used only a 0.5 mL (300 mcg/mL) dosage of G-CSF via the IU and SC routes before fresh ET in RIF patients with significant improvements in reproductive outcomes^26,29^. Other studies have used a 1 mL (300 mcg/mL) dosage via both routes with significant improvements in reproductive outcomes as well^27,28^. Both dosages (0.5mL and 1mL) were again used via IU and SC routes before frozen-thawed ET with significant improvement in reproductive outcomes^31,32^. Meta-analyses have analyzed these dosages and the reproductive outcomes. However, they have not given specific dose recommendations^17,33,37,39^. Therefore, we do not believe that using the higher volume dosage (1mL instead of 0.5mL) in our study have significantly influenced the outcomes. In our study, we used the 1mL (300mcg/mL) dosage and not the 0.5mL (300mcg/mL) as we found this dose is more commonly used in literature^17,33,37,39^. However, since significant results have been reported with the 0.5mL dosage, using a 0.5mL dosage in our study might have given the same results with less financial costs.

### Limitations and potential impact on results

Our study has a few limitations. First, we did not follow up the patients in either group to the time of delivery, and so, we could not evaluate the live birth rates, second-trimester abortion rates, and adverse pregnancy outcomes. As a fertility unit, we do not provide antenatal care, and our mission ends with a viable IU fetus by ultrasound two or four weeks after the positive pregnancy test. In our study, we followed up the patients to the end of the first trimester to assess the first-trimester abortion rate. Second, even though there was an increase in the endometrial thickness after G-CSF injection in the study group, we could not assess the significance of this outcome, as this information was rarely available in the control group due to poor documentation at our unit during the time before our study started. Third, we did not perform karyotyping for male and female partners or preimplantation genetic testing for aneuploidies [PGT-A] for embryos before ET. In patients with RIF, karyotyping both partners and preimplantation genetic screening should be considered or offered^7,11^. However, this is not available at public sector hospitals in our country, which hardly make basic IVF services available at a free or minimum charge to the infertile population. Finally, following our unit protocol at the initiation of the study, ET in our study was done on day 3 (cleavage stage) and not on day 5 (blastocyst stage). Blastocyst stage ET has increasingly been used in today’s IVF practice owing to a higher live birth rate than cleavage stage ET^7^. Also, recently, it has been considered a “can be considered” intervention by the ESHRE recommendations statement for RIF patients^7^.

It maybe worth noting that the study time interval was extended twice to meet the required sample size. This was due to the meticulous assessment of the study participants and ensuring that they met the eligibility criteria. Also, we encountered high ET cancellation rates in the study group after OPU and receiving the G-CSF treatment. A major part of this was due to frequent loss of embryos that was caused by interrupted power outages following an economic collapse in our country after the Ukraine war, with subsequent suspension of work for a few months until this was resolved. In addition, work was suspended at our unit for a year-long period during the Coronavirus disease 2019 [COVID-19] pandemic and is suspended yearly for the entire month of Ramadan. Finally, other interventional studies being conducted at our unit were also recruiting RIF patients, so we were unable to include these patients in our study. All these factors have led us to convert our study from a randomised clinical trial to a cohort study, in which we included all eligible patients in the study group and used data from previous patients who did not receive any treatment as the control group.

### Implications for future research| how to explore further

As the Subcutaneous route of administration was proven to be the most effective way of administering G-CSF (33,35,39), future studies may help to conclude whether G-CSF treatment before frozen-thawed or fresh ET in RIF patients is more effective. Future studies may also use a common definition to identify RIF patients, which can help reduce the heterogeneity and generate strong evidence in identifying RIF patients who will most benefit from G-CSF treatment.

## Conclusion

According to our study, G-CSF use via the IU route in idiopathic RIF patients before undergoing fresh ET was associated with higher implantation rates versus placebo, which was statistically significant. It was also associated with higher clinical and chemical pregnancy and lower abortion rates, but these were not statistically significant. Further studies in similar clinical settings are needed to draw definite conclusions.

## Data Availability

The datasets used and/or analysed during the current study are available from the corresponding author on reasonable request.

## Abbreviations

IVF: In vitro fertilization
IU: Intrauterine
RIF: Recurrent implantation failure
ESHRE: European Society of Human Reproduction and Embryology
G-CSF: Granulocyte-colony stimulating factor
ET: Embryo transfer
IOO: Induction of ovulation
OPU: Ovum pick-up
HCG: Human chorionic gonadotropin
CPR: Clinical pregnancy rate
IR: Implantation rate
SC: Subcutaneous
